# Pulmonary pleomorphic carcinoma with pseudoprogression during nivolumab therapy and the usefulness of tumor markers: A case report

**DOI:** 10.1002/ccr3.1627

**Published:** 2018-05-29

**Authors:** Akihiro Yoshimura, Chieko Takumi, Taisuke Tsuji, Ryosuke Hamashima, Shinsuke Shiotsu, Tatsuya Yuba, Yoji Urata, Noriya Hiraoka

**Affiliations:** ^1^ Department of Respiratory Medicine Japanese Red Cross Kyoto Daiichi Hospital Kyoto Japan; ^2^ Department of Clinical Pathology Japanese Red Cross Kyoto Daiichi Hospital Kyoto Japan

**Keywords:** immune checkpoint inhibitor, nivolumab, pseudoprogression, pulmonary pleomorphic carcinoma, tumor marker

## Abstract

Pseudoprogression was reported as one of the unconventional responses during immune checkpoint inhibitor therapy. A 70‐year‐old man with pulmonary pleomorphic carcinoma received nivolumab therapy. Pleural effusion and pulmonary metastasis increased, however then shrank and serum cytokeratin 19 fragment levels decreased. Serum tumor marker might help to distinguish pseudoprogression.

## 1 INTRODUCTION

A 70‐year‐old man with pulmonary pleomorphic carcinoma received nivolumab. Pleural effusion and pulmonary metastasis increased over 6 cycles; however, they shrank over 13 cycles of continuous nivolumab therapy. We diagnosed with pseudoprogression of pulmonary pleomorphic carcinoma. Serum cytokeratin 19 fragment levels decreased and might be useful to distinguish pseudoprogression.

Nivolumab significantly improved progression‐free survival and overall survival compared with docetaxel as second‐line therapy for non‐small cell lung cancer (NSCLC).^1^, ^2^ Thus, nivolumab was first approved as an immune checkpoint inhibitor (ICI) for NSCLC. An ICI is noted not only for its therapeutic effect but also for its unconventional responses and immune‐related adverse events. Pseudoprogression has been reported as one of the unconventional responses during ICI therapy.^3^ Pseudoprogression is a phenomenon in which the tumor shrinks after significant tumor growth during ICI therapy; it is not true disease progression.^4^ Current chemotherapy is discontinued when computed tomography (CT) shows significant tumor growth.^5^ Therefore, it is important to distinguish between pseudoprogression and true disease progression. Herein, we describe a patient with pseudoprogression in pulmonary pleomorphic carcinoma (PPC) during nivolumab therapy.

## 2 CASE REPORT

**Figure 1:**
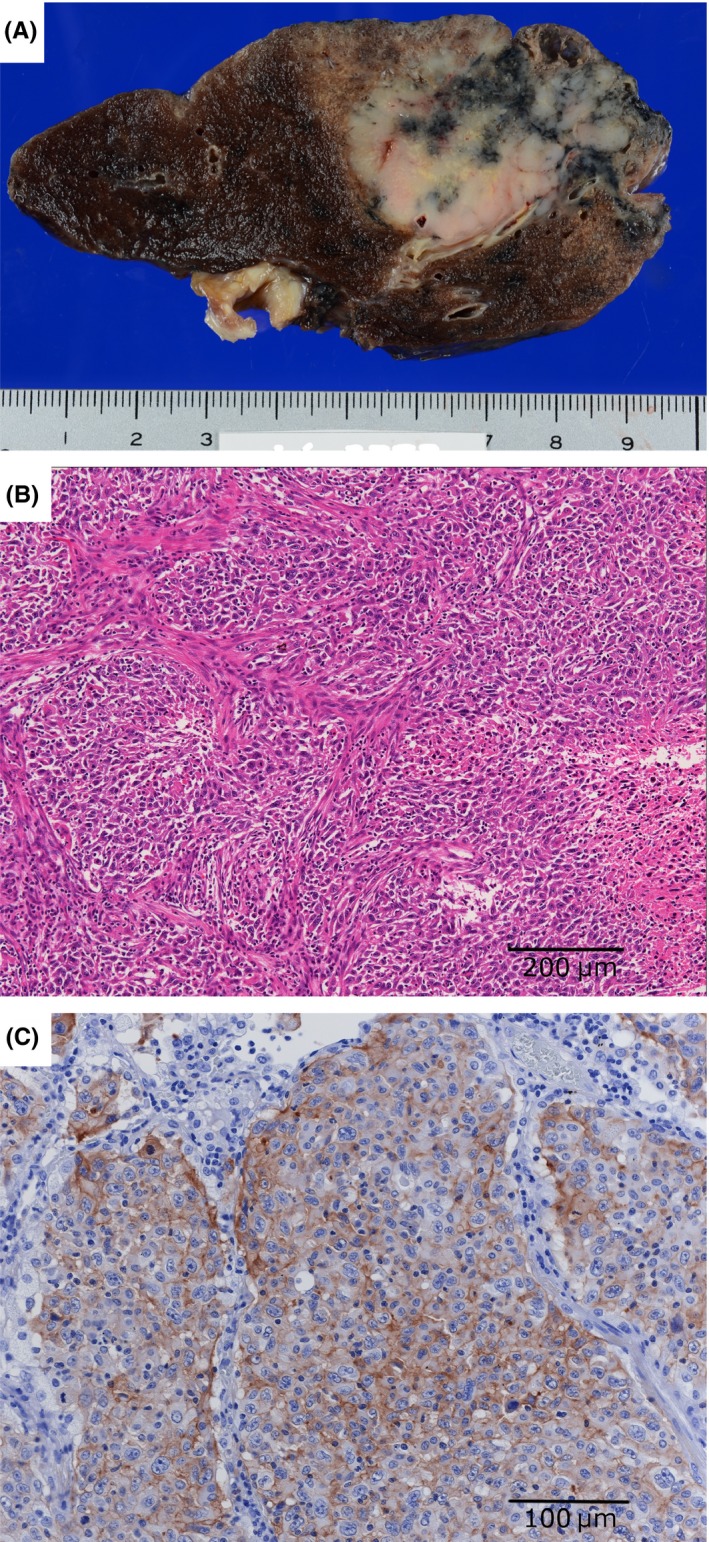
A, A yellowish‐white tumor with a size of 30 × 25 × 40 mm in the resected right lung. B, Hematoxylin and eosin staining (×100) detected pulmonary pleomorphic carcinoma with polygonal cells and spindle cells. C, Immunohistochemical analysis showed that 80% of tumor cells expressed programmed death ligand 1 (SP142; ×200)

**Figure 2:**
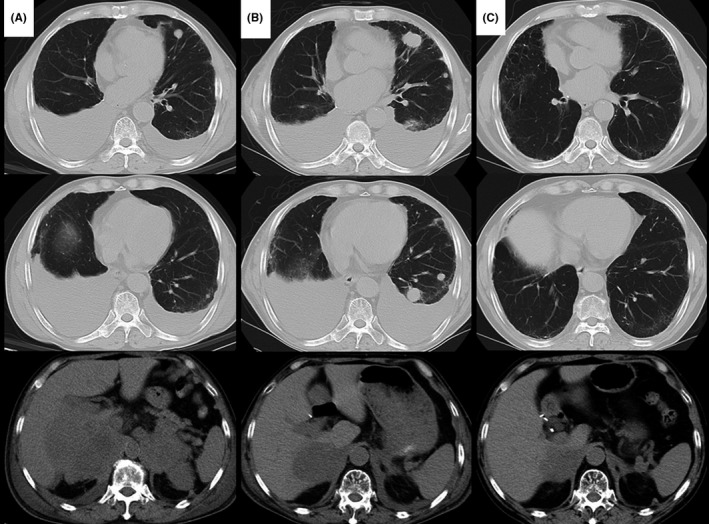
Computed tomography (CT) image during nivolumab therapy. A‐C, Before initiation, after 6 cycles, and after continuous administration of nivolumab therapy. CT showed that although the adrenal gland had shrunk without first increasing, the pleural effusion and pulmonary metastasis had increased once, and then shrunk

A 70‐year‐old man with a 50‐pack‐year smoking history was suspected to have lung cancer and underwent pulmonary resection of the right lower lobe in December 2014. He was diagnosed with PPC (Figure 1A,B; *EGFR/ALK*‐mutation negative) and pT2aN0M0 Stage IB (Union for International Cancer Control, UICC 7th edition) disease. Expression of programmed death ligand 1 (PD‐L1) was detected in 80% of the resected lung using an anti‐PD‐L1 SP142 antibody (Figure 1C). Although the patient had received adjuvant chemotherapy with tegafur/uracil, bilateral adrenal gland metastasis was detected in April 2015. We administered several chemotherapy regimens: carboplatin and paclitaxel (4 cycles); pemetrexed (9 cycles); vinorelbine (8 cycles); and docetaxel (2 cycles). Right adrenal metastasis increased and compressed the inferior vena cava, which caused leg swelling. At that time, CT revealed pleural effusion and pulmonary metastasis (Figure 2A). We administered nivolumab therapy as the fifth‐line therapy in October 2016. After 6 cycles of nivolumab therapy, the right adrenal gland metastasis was reduced, and his swollen legs and performance status improved; however, the pleural effusion and pulmonary metastasis were exacerbated (Figure 2B). The cytological analysis revealed that malignant cells were not detected and lymphocytes were predominant in pleural effusion. The left ventricular ejection fraction measured by echocardiography was 70% and the brain natriuretic peptide was 9.2 ng/mL (normal range < 18.4 ng/mL). The inferior vena cava (IVC) diameter was 20 × 8 mm and the respiratory variation in the IVC was more than 50%. Malignant pleural effusion and heart failure were unlikely. After 13 cycles, these lesions were improved by continuous nivolumab therapy (Figure 2C). Moreover, serum cytokeratin 19 fragment (CYFRA 21‐1) levels were 40.7 ng/dL (normal range <3.5 ng/mL) before the initiation of nivolumab therapy and they decreased to 8.7 ng/dL after 6 cycles and to 4.1 ng/dL after 13 cycles. Serum carcinoembryonic antigen (CEA) levels were 2.9 ng/mL (normal range <5.0 ng/mL) before the initiation, 2.6 ng/mL after 6 cycles, and 3.9 ng/mL after 13 cycles. We diagnosed the patient with pseudoprogression of PPC. Follow‐up is ongoing to date (June 2017), and he has achieved a partial response with continuous nivolumab therapy without exacerbation or adverse effects.

## 3 DISCUSSION

Immune checkpoint inhibitor therapy could cause a distinct immune‐related pattern of responses.^3^ Some patients were reported to present with tumor shrinkage after significant growth during ICI therapy.^4^ This finding is called pseudoprogression and has been reported to occur in 5.1%‐6.7% of patients with NSCLC during nivolumab therapy.^1^, ^2^ Pseudoprogression in PPC has also been reported;^6^ however, it is rare and has only been described in a few case reports. In addition to NSCLC, pseudoprogression has been reported to affect 6.7%‐12% of patients with malignant melanoma.^3^ The proposed mechanism of pseudoprogression is that T‐cells activated by ICI infiltrate the tumor; CT has shown that the tumor seems to become larger, even though the lesion ultimately shrinks.^7^

We constantly perform CT and use the Response Evaluation Criteria in Solid Tumors (RECIST) to evaluate the therapeutic effect. If the disease has progressed, we will consider alternative chemotherapy. This patient’s pleural effusion and pulmonary metastasis were exacerbated after 6 cycles of nivolumab therapy, which was accepted as disease progression according to the RECIST. However, these lesions were improved by continuous nivolumab therapy. As a result, these findings were not true disease progression and were instead pseudoprogression. There is no criterion for pseudoprogression. However, evaluating the patient’s general condition and performance status has been reported as a useful method to distinguish between pseudoprogression and true disease progression.^3^, ^5^ In pseudoprogression, a patient’s general condition improves and the CT shows that the tumor shrinks after increasing once, which was consistent with the findings in our patient. In addition, his serum CYFRA 21‐1 levels decreased at the time of significant tumor growth during nivolumab therapy, while serum CEA levels did not change. Although there is a relationship between PPC and serum tumor marker levels, serum CYFRA 21‐1 levels decreased in NSCLC and this has been reported to be a superior marker compared with serum CEA levels of the response to chemotherapy.^8^ CYFRA 21‐1 might be one of the indicators of pseudoprogression in PPC. However, in a past case report,^9^ serum CEA levels decreased in patients with pseudoprogression during nivolumab therapy and further study is needed. Moreover, in this patient, we did not measure tumor marker levels in the first months after nivolumab administration; therefore, we could not observe how these levels changed over time, which may provide further insight into their clinical benefit.

PPC is classified as an NSCLC sarcomatoid carcinoma^10^; PPC is composed of spindle cell, multinucleated cell, and giant cell carcinomas. PPC predominantly affects male smokers, and is a rare disease with a poor prognosis.^11^ Recurrent PPC has a poor response to chemotherapy and effective treatment is needed. Kanazu et al^12^ reported that nivolumab is effective for PPC in a case series. Although nivolumab therapy is not currently a standard therapy for PPC, it is expected to become an important therapy for PPC in the future. In the present case, the patient achieved a partial response even with pseudoprogression. We used this antibody clone because, at the time of encountering this case, the guidelines recommending the use of the 28‐8 and 22C3 clones had not been published. Furthermore, the SP142 assay was more cost‐effective in this case.

In conclusion, we encountered a PPC patient with pseudoprogression during nivolumab therapy. The patient’s tumor marker levels decreased even during pseudoprogression. Tumor markers might be useful to distinguish pseudoprogression from true disease progression. Further studies are needed to investigate pseudoprogression mechanisms. Measuring tumor markers routinely is important in addition to evaluating the patient’s general condition and performance status during nivolumab therapy for PPC.

## CONFLICTS OF INTEREST

None declared.

## AUTHORSHIP

AY: wrote the manuscript and created the Figure. CT: treated the patient. TT: wrote the manuscript. RH: treated the patient. SS and TY: discussed the diagnosis. YU: provided pathological comments. NH: provided clinical comments.
